# General and anxiety-linked influences of acute serotonin reuptake inhibition on neural responses associated with attended visceral sensation

**DOI:** 10.1038/s41398-024-02971-3

**Published:** 2024-06-06

**Authors:** James J. A. Livermore, Lina I. Skora, Kristian Adamatzky, Sarah N. Garfinkel, Hugo D. Critchley, Daniel Campbell-Meiklejohn

**Affiliations:** 1 School of Psychology, University of Sussex, Brighton, UK; 2 Heinrich Heine Universität, Düsseldorf, Germany; 3 Sussex Centre for Consciousness Science, University of Sussex, Brighton, UK; 4 Institute of Cognitive Neuroscience, University College London, London, UK; 5 Brighton and Sussex Medical School, Brighton, UK; 6 Sussex Partnership NHS Foundation Trust, Brighton, UK

**Keywords:** Neuroscience, Human behaviour

## Abstract

Ordinary sensations from inside the body are important causes and consequences of our affective states and behaviour, yet the roles of neurotransmitters in interoceptive processing have been unclear. With a within-subjects design, this experiment tested the impacts of acute increases of endogenous extracellular serotonin on the neural processing of attended internal sensations and the links of these effects to anxiety using a selective serotonin reuptake inhibitor (SSRI) (20 mg citalopram) and a placebo. Twenty-one healthy volunteers (fourteen female, mean age 23.9) completed the Visceral Interoceptive Attention (VIA) task while undergoing functional magnetic resonance imaging (fMRI) with each treatment. The VIA task required focused attention on the heart, stomach, or visual sensation. The relative neural interoceptive responses to heart sensation [heart *minus* visual attention] (heart-IR) and stomach sensation [stomach *minus* visual attention] (stomach-IR) were compared between treatments. Visual attention subtraction controlled for the general effects of citalopram on sensory processing. Citalopram was associated with lower interoceptive processing in viscerosensory (the stomach-IR of bilateral posterior insular cortex) and integrative/affective (the stomach-IR and heart-IR of bilateral amygdala) components of interoceptive neural pathways. In anterior insular cortex, citalopram reductions of heart-IR depended on anxiety levels, removing a previously known association between anxiety and the region’s response to attended heart sensation observed with placebo. Preliminary post hoc analysis indicated that citalopram effects on the stomach-IR of the amygdalae corresponded to acute anxiety changes. This direct evidence of general and anxiety-linked serotonergic influence on neural interoceptive processes advances our understanding of interoception, its regulation, and anxiety.

## Introduction

If asked how one feels, it is natural to turn attention to the body. This involves interoception—the sensing and processing of internal physiological states [1–3]. The influence of ordinary interoception ranges from basic regulatory reflexes that maintain life with homoeostasis to the conscious affective experiences of hunger, arousal, anxiety, and self that can impact mental health [2, 4–7]. Yet, little is known about the pharmacology of ordinary interoception or how this pharmacology determines and is determined by affective states. Here, we studied the impact of acute changes of serotonin on ordinary interoception and how this influence relates to anxiety.

Serotonin is recognised for its ability to acutely modulate exteroceptive sensory processing, such as hearing and vision, according to the animal’s needs and environment. In this context, increased serotonin activity is proposed to have a nuanced tempering effect on the flow of sensory information [8, 9].

Serotonin has also been shown to modulate interoception when sensations are aversive and exogenous, such as when a balloon or acid is applied within the digestive tract [10–15]. These effects can depend on the stimulation site and participant states of sensitivity and perception of pain. In general, however, single doses of selective serotonin reuptake inhibitors (SSRIs) tend to reduce the sensitivity and aversiveness of exogenous interoception of the digestive tract while depleting serotonin’s precursor tends to amplify it. Whether or not serotonin’s influence extends to interoception of ordinary endogenous gastric sensations is not yet clear.

Similarly, little is understood about serotonin’s role in the interoception of the heart. In a behavioural study of healthy volunteers, a single SSRI dose increased metacognitive insight into the interoception of heartbeats [16]. In major depression, patients tend to have blunted interoceptive processing of the heart and stomach [17]. Patients with depression receiving chronic SSRI treatment have demonstrated higher subjective interoceptive intensities than patients not receiving SSRI treatment [18]. However, the neural correlates of these SSRI effects have remained unclear.

An association between serotonin and ordinary interoception might be expected because both are commonly associated with anxiety. The serotonin-anxiety relationship has been established by considerable preclinical research [19–21] and SSRI effectiveness as a first-line pharmacological treatment of panic and generalised anxiety disorder [22, 23]. SSRIs can also increase anxiety in the short term [24, 25]. The interoception-anxiety relationship is characterised by a theoretical understanding of the role of interoception in the pathoaetiology and treatment of somatic anxiety symptoms and backed by empirical observations of interoceptive disturbance in the same anxiety disorders that are treated with SSRIs [7, 26–28]. Modern conceptualisations of irritable bowel syndrome also acknowledge neural, genetic, homoeostatic and pharmacological overlap between gastrointestinal sensation and anxious states [29]. In the lab, many of the same anxiety-linked responses that SSRIs acutely influence will vary with interoceptive sensation—including startle [30–33], fear [34, 35], and emotion recognition [36–39]. Conversely, SSRI treatment can also cause emotional blunting, which could be attributable to the general suppression of interoceptive processes, consistent with effects on aversive gastric interoception [15], or a change in interoception’s influence on subjective affective experience [40–42].

The neural substrates of interoception, anxiety, and serotoninergic influence on cognition also overlap. At serotonin terminals, this occurs within the insular cortex and amygdala [3, 17, 20, 29, 39, 43–50]. However, serotonin’s interoceptive effects could also occur at cell bodies within the raphe nuclei. The raphe nuclei contain cells that modulate appetitive, cardiac, respiratory, sensory, and thermal regulatory processes in response to interoceptive information [51, 52] and are thought to play a critical role in regulating anxiety [53]. SSRIs disproportionately increase extracellular serotonin at the raphe nuclei following acute doses [54].

Overall, our knowledge about the relationship of serotonin to ordinary interoceptive processing has been promising but limited by the testing of abnormal, painful, visceral sensations in small samples, the study of patient populations with disturbed interoception, absent exteroceptive control conditions, indirect inferences and lack of neural insight [55]. No direct link of serotonin to the neural processing of ordinary interoceptive sensation has been established.

Critically, some effects of serotonin on interoception are likely to be state-dependent. Serotonergic inhibitory effects on exteroceptive processing [9, 56, 57] and exogenous gastrointestinal interoception [10, 11, 14] are sensitive to the environment and state of the individual. This and other findings suggest that serotonin could constrain the impact of these states on emotion, cognition and behaviour through the regulation of sensory information processing [58]. One state of particular interest for interoception and serotonin is anxiety. The anterior insular cortex responds more to attended cardiac sensation in anxious individuals, and the cognition of anxious individuals can be more susceptible to interoceptive influence [44, 45, 59]. As such, we considered that some influences of serotonin on interoception might be particularly evident during anxious states and thereby modulate the interoception-anxiety relationship. Previously, the selectivity of serotonin effects for anxious states has been observed in the amygdala, wherein a single SSRI dose reduced the enhanced behavioural and neural response to fearful faces in relatively anxious individuals (also with past or present depression) but not in less-anxious, healthy controls [60, 61].

Neural responses to ordinary endogenous interoception can be probed by asking individuals to shift and maintain attention to a specific visceral sensation, which typically enhances the regional neural response underlying the corresponding interoceptive representation [17, 18, 62–65]. Neural responses to different interoceptive sensations have different implications for health and behaviour [2, 29, 59, 62]. Attended heart and stomach sensations have been shown to have overlapping but ultimately distinct representations in the human brain [66]. Correspondingly, researchers have examined the neural correlates of cardiac and gastric interoception separately within the VIA task in various clinical contexts after subtracting effects on visual attention to control for general effects on sensory processing [17, 18, 62–65]. We combined this approach with the established pharmacological technique of using a single SSRI dose in healthy volunteers [39, 50] to test the influence of increased extracellular serotonin on heart and stomach interoceptive processing. While long-term SSRI doses are essential for modelling the mechanisms of their delayed therapeutic effects, acute dose studies provide insight into the impacts of immediate serotonin changes on cognition without confounds of receptor desensitisation and neuroplasticity theoretically linked to long-term SSRI treatment [54, 67]. Single-dose studies can also provide needed mechanistic insight into early side effects of SSRIs, such as anxiety [25], and the immediate effects of SSRIs on affective bias that could theoretically contribute to and predict long-term clinical outcomes [39]. Studies of healthy volunteers avoid extraneous confounds associated with disorders and allow for a powerful within-subject analysis without requiring a placebo control in patients who would benefit from medication.

We recognised that the influence of SSRIs could be expressed in different ways across the hierarchical network of interoceptive substrates, which change in function from supporting basic interoceptive sensory representations (e.g. mid-posterior insular cortex) to their integration into motivational and affective states (e.g. anterior insular cortex and amygdala) [3]. We, therefore, tested two hypotheses across these networks. Considering that serotonin tends to generally inhibit exteroceptive processing [8, 56–58], reduce visceral pain [10, 14, 15], increase emotional ‘blunting’ [40, 68], reduce responses to rewards and punishments [69], and reduce amygdala and insula responses to cues of threat [70]—the primary hypothesis was that an acute SSRI dose would attenuate the general neural response to ordinary internal sensations. The secondary hypothesis was that some SSRI effects on interoception would be selective for anxious states. We then undertook a post hoc exploration of associations between acute SSRI effects on interoception and changes in anxiety.

## Methods and materials

### Experimental design

We completed a placebo-controlled, double-blind, randomised cross-over, pharmacological neuroimaging experiment whereby the same participants were tested in two sessions, one following a single dose of an SSRI and one following a single placebo, in random order. The SSRI (20 mg oral citalopram (Cipramil, Lundbeck Ltd, Watford, UK) was chosen for its common use, its safety profile, and its exceptionally high selectivity for the serotonin transporter [71].

### Participants

Ethical permission was granted by The Brighton and Sussex Medical School Research Governance and Ethics Committee (ER/JL332/9). Potential participants were screened with a health questionnaire and a standard psychiatric interview. Each participant provided informed consent. Exclusion criteria included: age under 18 years or over 35 years; the presence of significant ongoing medical condition; pregnancy or breastfeeding; currently taking any medication (excluding contraceptive pill); first-degree family history of bipolar disorder; an indication of current or historical mental health disorder, or scanner contraindications (e.g. metallic implants). Participants were instructed to abstain from alcohol or caffeine in the preceding 12 h before the start of test sessions.

Thirty-one healthy participants were recruited for the study and randomised to a treatment sequence. The sample size was determined by available funding, exceedance of sample sizes used to establish prior acute citalopram effects on neural responses to affective cognition [39], our within-subject design, and the probable 30% attrition rate. Seven participants withdrew from the study. Neuroimaging data from two participants were excluded due to excessive motion ( > 6% of volumes identified as motion outliers for either session scan). Neuroimaging data for one participant was excluded due to extreme nausea requiring exiting of the scanner. This left 21 participants measured in each condition, with 42 sessions in total across both treatments. This sample (mean age of 23.9 (*SD* = 3.3); and a mean body mass index of 21.9 (*SD* = 3.3)) included seven females. Trait anxiety scores (State-Trait Anxiety Inventory [72] (STAI)) averaged 34.9 (*SD* = 8.3). Eleven participants lived with low trait anxiety (score below 35), nine participants lived with moderate trait anxiety (score between 35 and 44), and one participant lived with high trait anxiety (score of 50). As measured by the Body Perception Questionnaire [73], the sample’s interoceptive tendencies fell within the normal range: their awareness subscale mean was 2.4 (*SD* = 1.0), and the stress subscale mean was 2.5 (*SD* = 0.88).

### Study procedure

Participants were tested twice, with separate citalopram and placebo sessions, at least seven days apart (*M* = 10.3 days, *SD* = 6.87). The assignment of participants to treatment order was completed by a researcher with no contact with participants and no awareness of participant identities, using a computer-based algorithm for randomisation. Participants and researchers in contact with participants were blind to treatment conditions. Citalopram and placebo doses were contained in identical gelatine capsules filled with microcrystalline cellulose by a pharmacist.

After the dose, participants were given instructions and time to practice the VIA task before testing. Just before scanning, heart rate was recorded with the participant relaxed and sitting. Heart rate variability was measured as the standard deviation of the pulse interval. To allow citalopram levels to reach peak absorption, participants performed the VIA task approximately 3.75 h after treatment intake.

State anxiety (STAI-S) was measured immediately before and after the scan session. We used the average of the two measurements to estimate anxiety in the scanner. Similarly, to relate to prior studies and capture any overall change in mood, participant affect was measured with the Positive and Negative Affect Scale (PANAS) before and after scan sessions. The PANAS includes features of state anxiety in addition to other measures of affect. So, only the STAI-S was used to test hypotheses related to anxiety. Participants also completed eight visual analogue scales before and after the scan (the average of these taken for analysis, estimating scanner experiences). Three scales (from 0 to 100) were given to assess three somatic side effects (nausea, headache and dizziness). Five anxiety-related effects (pairs of antonyms: alert−drowsy, stimulated−sedated, restless−peaceful, irritable-good-humoured, anxious−calm) were used to confirm the STAI-S measures and alert the researchers to excessive side effects.

### Visceral interoceptive awareness task

**Fig. 1:**
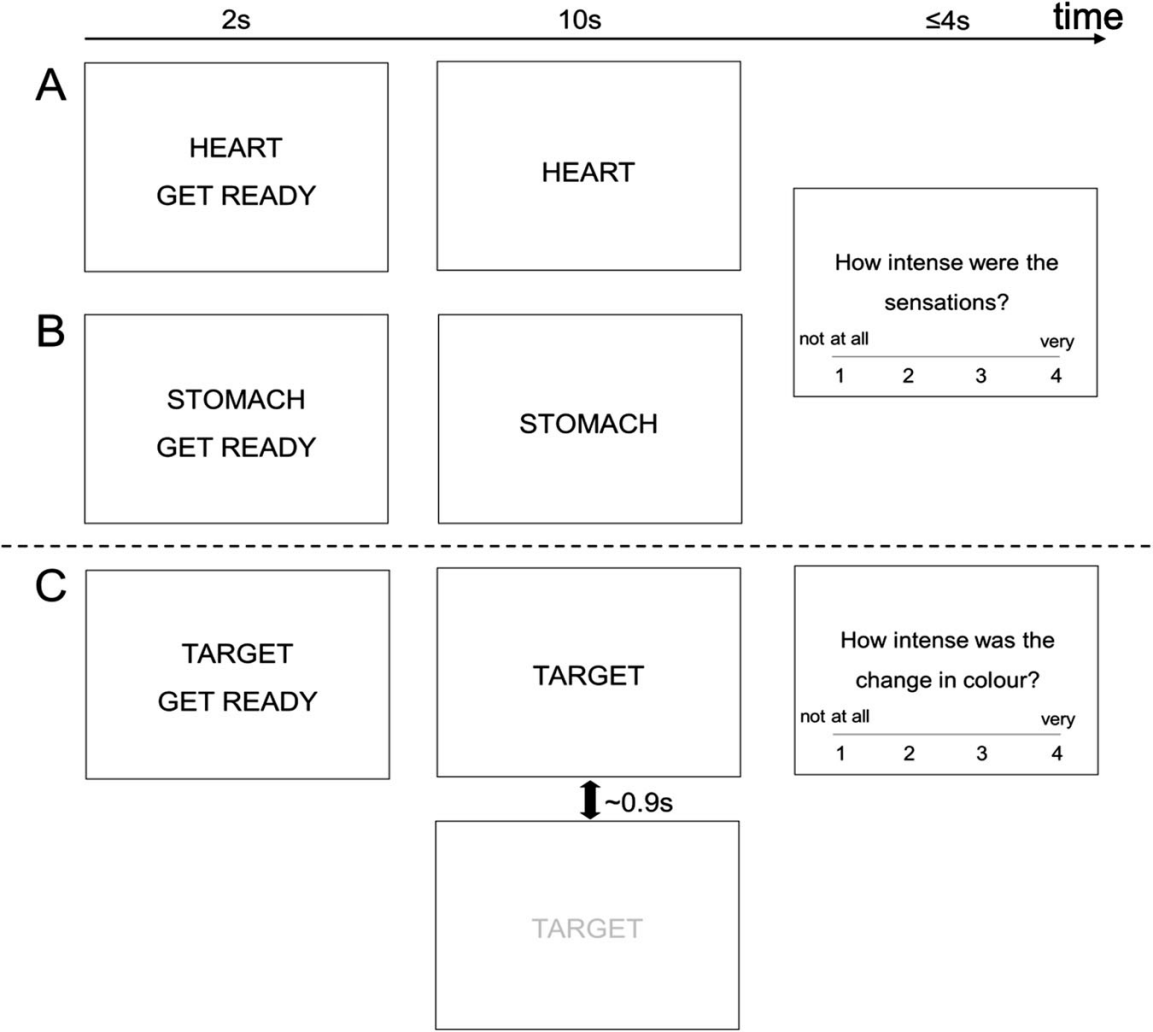
Visceral Interoceptive Attention Task. Participants focus on **A** heart, **B** stomach, and **C** exteroceptive sensation. Each condition was presented 15 times. The rating phase was presented eight times for conditions **A** and **B** and seven times for condition **C**.

The VIA task (Fig. 1) has been used previously to identify changes in neural interoceptive processing in multiple clinical contexts [17, 18, 62–65]. The two interoceptive conditions consist of the *Heart* and *Stomach* cue trials, and the exteroceptive (visual) baseline condition consists of *Target* trials. In the *Target* trials, after a short interval, the target started changing from black to grey and cycled between these shades for a randomly varied time between 0.7 s and 1.1 s, designed to mimic the approximate frequency of a heartbeat. In approximately half the trials, participants were asked to rate the intensity of the sensations from the area of focus (interoceptive trials) or the intensity of the colour change (exteroceptive trials). These responses helped to ensure that participants focused but were too infrequent for statistical inference. The trials selected for intensity rating were the same for every participant in a randomised sequence. Sensory condition order (*Heart, Stomach, Target*) and shades of grey for the *Target* blocks were presented in the same randomised sequence for every participant. There were 15 trials in each condition for a total task duration of 15 min.

Participants were given the following instructions:

‘While the word ‘HEART’ or ‘STOMACH’ is shown on the screen, focus attention on the intensity of the sensations experienced from the area of your heart or stomach. When the word ‘TARGET’ is shown on the screen, it will sometimes change from black to different shades of grey. Focus attention on the amount that the colour changes. When a CROSS is shown on the screen, you can rest. Please keep your eyes open and try not to think of anything in particular. You will sometimes be asked to rate how intense the sensations or the colour changes were, using the buttons shown. Please make sure that you breathe evenly throughout the task. You must NOT hold your breath at any time.’

### Neuroimaging procedures

Magnetic resonance imaging data were acquired using a Siemens Magnetom Prisma 3T scanner (Siemens Healthcare GmbH, Erlangen, Germany) and a 32-channel head coil. We acquired fMRI images during VIA task performance, field maps, perfusion images to test for treatment effects on cerebral blood flow (see Supplemental Material) and T1-weighted structural images for registration.

### Image acquisition

fMRI images were acquired using T2*-weighted echo-planar imaging sensitive to blood oxygenation level-dependent signal changes (multiband factor 4, echo time 37 ms, repetition time 1500 ms, voxel size 2.2 × 2.2 × 2 mm, 104 × 104 voxels per slice, 72 slices, field of view (FOV) 205 × 205 mm^2^, flip angle 52°), with a varying number of volumes according to the speed of task completion (*M* = 580, *SD* = 36.8). Before the task fMRI sequence on both test sessions, pairs of phase-encode reversed images were acquired for distortion correction (FOV 228 × 228 mm^2^ 104 × 104 voxels per slice, echo spacing 0.54 ms). In one session, a T1-weighted magnetisation-prepared rapid acquisition gradient echo (MPRAGE) structural image of the brain was acquired (echo time 2.2 ms, repetition time 2400 ms, 0.8 mm isotropic voxels, 300 × 320 voxels per slice, 208 slices, FOV 256 × 256 mm^2^, flip angle 8°).

### Pre-processing

fMRI images were pre-processed with a standard FMRIB Software Library v. 6.02 (FSL) [74] pipeline including motion correction using the middle volume of the time series as a reference volume, identification of motion outlying volumes using FSL Motion Outliers (root mean squared intensity difference of adjacent volumes method), distortion correction using phase-encode reversed image pairs (using FSL tool ‘topup’), high-pass filtering at 80 s, brain extraction, and co-registration to participant structural images using linear registration. Registration from structural space to standard Montreal Neurological Institute space (MNI152 nonlinear 6^th^ generation) was carried out using nonlinear registration. Independent Component Analysis (ICA) decomposition [75] was completed on unsmoothed data, and denoising was carried out manually, with noise components identified by an independent researcher using published criteria [76]. Spatial smoothing was completed with a 5 mm full-width at half maximum Gaussian kernel.

### Analyses

Treatment differences in self-reported sensation intensities during the VIA task, heart rate, STAI-S and PANAS scores and VAS scales were analysed using paired t-tests.

Functional images were analysed using FSL’s FMRI Expert Analysis Tool (FEAT) v 6.0 with two-level generalised linear models (GLMs). The first-level models corresponded to individual scan sessions. These included events corresponding to trial conditions (*Heart*, *Stomach* and *Target*) along with regressors of no interest: intensity rating periods, button presses, outlying motion volumes and temporal derivatives of each event. Neural responses to exteroceptive focus (*Target*) served as high-level controls. They were subtracted from responses to interoceptive focus (*Heart*, *Stomach*) to generate contrast images of the relative interoceptive response to heart focus (heart-IR) and stomach focus (stomach-IR). The subtraction of exteroceptive effects controls for general treatment effects on sensory processing.

We completed three second-level analyses. Hypothesis 1 was tested by comparing heart-IR and stomach-IR between treatment conditions. This used FSL’s paired two-group difference design (i.e., a multilevel mixed effect model with a random intercept) with FMRIB’s Local Analysis of Mixed Effects (FLAME) 1 & 2 over the heart-IR and stomach-IR contrasts of all 42 scan sessions. Fixed factors were treatment (placebo, citalopram) and test session (1st or 2nd). Hypothesis 2 was tested by a second-level model designed to test for anxiety-dependent effects and how the relationship between anxiety and interoceptive response (heart-IR and stomach-IR) changed on citalopram. This was like the first model but added centred STAI-S score (which can vary between sessions within subjects) and anxiety-by-treatment interaction (STAI-S scores multiplied by +1 for citalopram and −1 for placebo) regressors. A drug-induced change in the relationship between anxiety and relative interoceptive response was tested by separate one-sample t-tests over the anxiety-by-treatment interaction for heart-IRs and stomach-IRs across all 42 sessions. To confirm that a significant anxiety-by-treatment interaction effect (observed for heart-IR) changed an existing baseline unmedicated relationship between anxiety and heart-IR (without citalopram), replicating prior studies, a third whole-brain search was conducted across all participants in the placebo condition, using regressors of the intercept, test session, and mean-centred STAI-S scores. A one-sample t-test was completed across the latter.

Unless otherwise specified, all reported findings at the second level were whole-brain searches, cluster-corrected using a conservative voxel-wise threshold of Z > 3.1 and a cluster significance level of *p* < 0.05. However, we applied small volume correction where a whole-brain search failed in the main heart-IR comparison. Since we had strong predictions that effects would be observed in or around the amygdala [50] and insular cortex [17], we generated masks for these regions with the Oxford-Harvard cortical and subcortical probabilistic atlases (using any probability of including the designated region). Only two masks were tested: bilateral amygdalae and bilateral insular cortices (including nearby planum temporale, Heschl’s Gyrus, and operculum).

Order effects were tested independently to establish no confounding effects of the session or session × treatment interaction.

Post-hoc, the six significant clusters from the treatment effects of the first second-level model (Hypothesis 1) were tested for association with increases and decreases of anxiety between sessions after controlling for anxiety-associated side effects and treatment order. Criteria for reporting was either a significance level beyond a Bonferroni corrected threshold (*p* < 0.0083) or a similar effect in the same region on the other side of the brain, which would be unlikely to occur by chance.

Supplemental tests examined the robustness of effects against side effects of the treatments, treatment order, changes in cerebral blood flow, and guess of treatment condition. A separate supplemental regression was also conducted between treatment effects on cardiac interoceptive awareness reported elsewhere [16] and treatment effects on heart-IR. Correlations between clusters were explored. A separate regression between trait anxiety and citalopram’s effect on heart-RIR was also conducted. For all of the above, please see Supplemental Material.

Data associated with this project can be freely downloaded at 10.6084/m9.figshare.22786274

## Results

### Heart rate and subjective experience

Heart rate was lower on citalopram (*M* = 63.1 bpm, *SD* = 8.44) than on placebo (*M* = 66.53 bpm, *SD* = 10.6), consistent with the effective absorption of citalopram (t(20) = −2.45, *p* = 0.02) [77]. There was no change in a specific recorded subjective experience. However, participants rated the likelihood of having received citalopram higher (t(20) = 2.42, *p* = 0.03) on a scale of 1–100 when they had received citalopram (*M* = 56.9, *SD* = 22) compared to placebo (*M* = 32.0, *SD* = 22), indicating a potential summative influence of subjective effects on conscious awareness of the drug condition. See Supplemental Material for a thorough investigation into the robustness of results relating to this effect. See Table S1 for all measures of heart rate and subjective experience.

### Cerebral blood flow

Analysis of ASL image pairs (Supplemental Material) showed no significant clusters at the familywise error rate, suggesting that citalopram did not affect cerebral blood flow. Therefore, any effects on BOLD responses were unlikely to be mediated by general effects on blood flow in the brain.

### Interoceptive Attention: fMRI

**Fig. 2:**
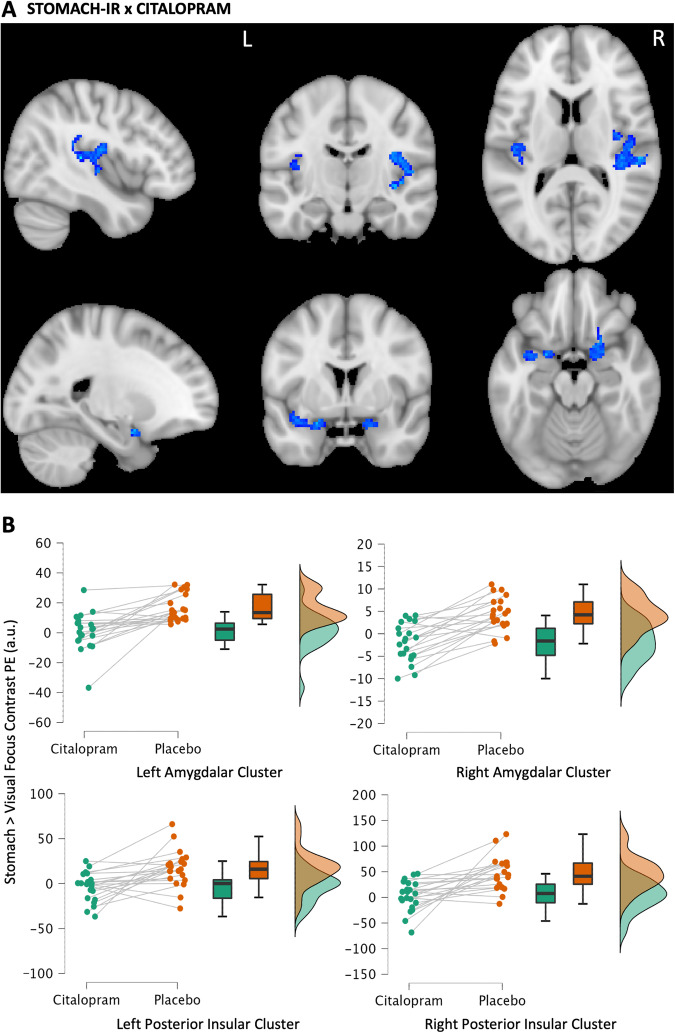
Citalopram Effect on the Interoceptive Response to Attended Stomach Sensation. **A** Reduced activity (blue) of the stomach-IR with citalopram (vs. placebo). Maps were developed with a voxel threshold of Z > 3.1 and a cluster significance threshold of *p* < 0.05. Maps are overlayed onto the standard MNI 152 brain (x, y, z coord: top 43,−15, 12; bottom −22, 2, −18). R and L indicate right and left. **B** Plots of the average contrast parameter estimates (PE, arbitrary units) for each significant cluster in A. Green = citalopram. Orange = placebo.

**Fig. 3:**
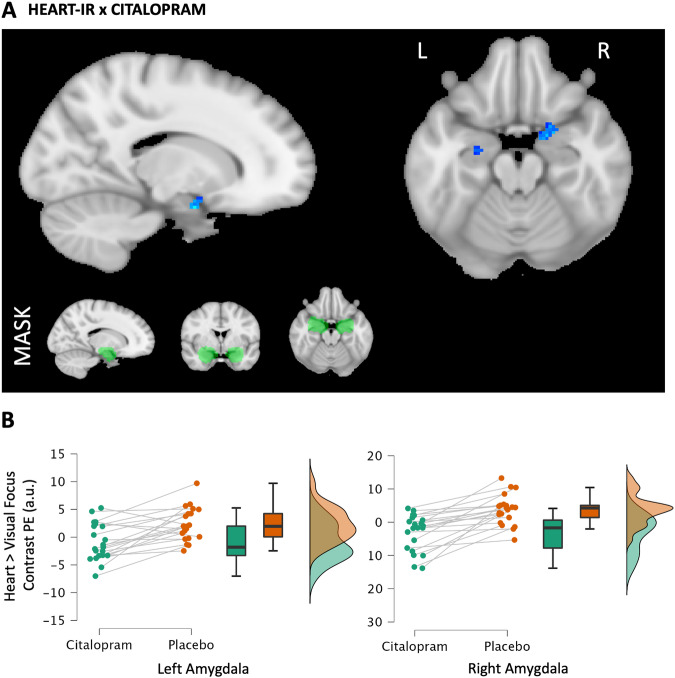
Citalopram Effect on the Interoceptive Response to Attended Heart Sensation. **A** Reduced amygdala activity (blue) of the heart-IR with citalopram (vs. placebo). Maps were developed with a voxel threshold of Z > 3.1, a cluster significance threshold of *p* < 0.05, and small volume correction within an anatomical mask (light green) of amygdalae from the Harvard-Oxford Subcortical Structural Atlas. Maps are overlayed onto the standard MNI 152 brain (x = 16, z = −18). **B** Plots of the average contrast parameter estimates for heart-IR (PE, arbitrary units) in each treatment condition for each significant cluster in A. Green = citalopram. Orange = placebo.

Relative to placebo, citalopram reduced the relative interoceptive response to stomach sensation (stomach-IR) within the amygdalae, bilateral posterior insular cortex, and neighbouring regions (Table 1, Fig. 2) using a whole brain search. These are referred to henceforth as (left and right) *amygdalar* and *posterior insular* clusters, respectively. During heart focus, treatment differences did not reach the same threshold as the stomach focus when correcting for multiple comparisons across the whole brain. Using small volume correction, citalopram was shown to reduce the heart-IR within the right and left amygdalae (Table 1, Fig. 3). However, there was no evidence for a similar reduction in the insular cortices. A separate analysis demonstrated no order effects within suprathreshold clusters of BOLD activity affected by citalopram.

**Table 1:** fMRI Effect Clusters.

Contrast and Anatomy	Voxels	Z MAX	X	Y	Z
**Citalopram Effect on Stomach-IR**					
right posterior insular cluster: planum temporale peak extending to Heschl’s gyrus, central operculum and posterior insular cortex	609	4.85	44	−30	10
left posterior insular cluster: parietal opercular cortex peak extending to Heschl’s gyrus, planum temporale, central operculum and posterior insular cortex	155	4.35	−38	−30	20
left amygdalar cluster: amygdala peak extending to the anterior insular cortex, parahippocampal gyrus	135	4.35	−22	2	−18
right amygdalar cluster: frontal orbital cortex peak extending into the amygdala	107	4.23	20	8	−16
**Citalopram Effect on Heart-IR (within Amygdala Mask)**					
right amygdala, orbitofrontal cortex, parahippocampal gyrus	67	4.28	16	0	−22
left amygdala, hippocampus	23	3.74	−22	−10	−18
**Citalopram x State Anxiety Association with Heart-IR**					
frontal orbital cortex, insular cortex, inferior frontal gyrus	341	4.53	44	28	−8
occipital cortex	216	5.49	34	−88	−10
frontal orbital cortex, insular cortex	118	4.4	−38	24	−4
lateral, occipital cortex, precuneus	109	4.31	10	−82	48
occipital pole	86	5.22	−36	−94	4

Contrast, region (Oxford Harvard Cortical and Subcortical Atlas), number of voxels in cluster, peak voxel z score and peak coordinates (x,y,z) in MNI space.

### Anxiety

State anxiety decreased for some participants and increased for others following an acute dose of citalopram. This resulted in no average anxiety change (*p* > 0.72) (Table S1).

**Fig. 4:**
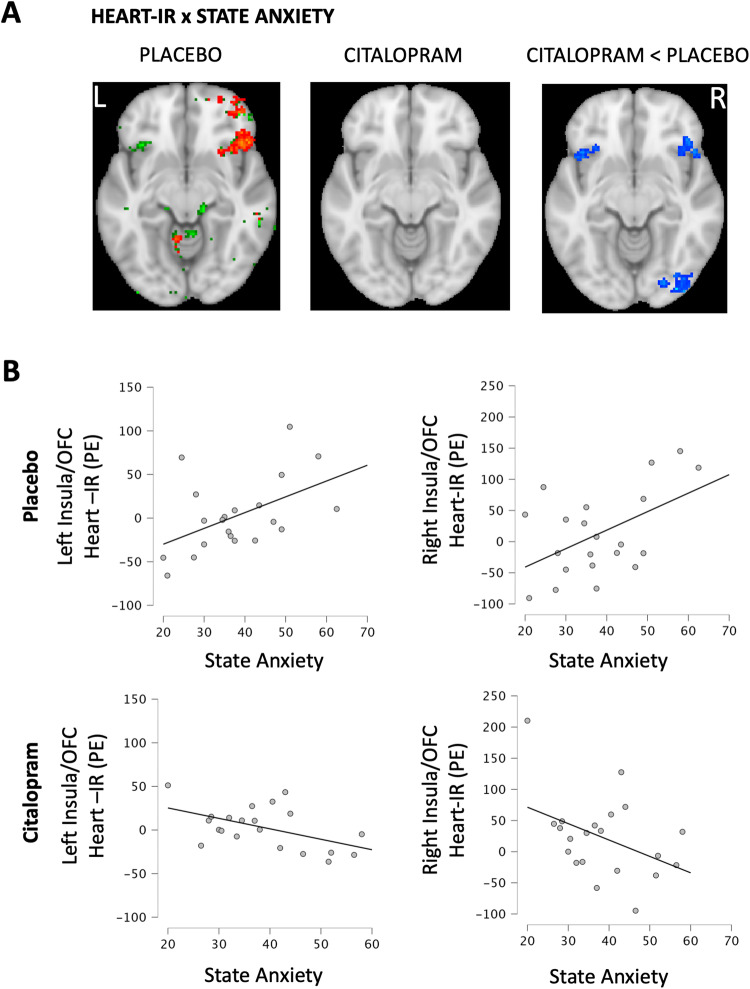
Citalopram Effect on the Anxiety-Interoception Relationship. **A** Positive association between heart-IR and state anxiety on placebo (red/green) and the significant reduction of this relationship on citalopram (blue). Red and blue maps result from whole-brain searches using a voxel threshold of Z > 3.1 and a cluster significance threshold of *p* < 0.05. Green maps result from a whole brain search, uncorrected at *p* < 0.001. Maps are overlayed onto the standard MNI 152 brain (z = −10). **B** Plots of state anxiety (STAI-S) associations with average heart-IR parameter estimates (PE, arbitrary units) within the frontal orbital cortex / insular clusters of the citalopram < placebo contrast of A. Refer to Table 1 for statistical inference.

In a whole-brain search, we discovered an effect of citalopram that increased with state anxiety within symmetrical bilateral clusters that spanned anterior insular and posterior orbitofrontal cortices (Table 1, Fig. 4A). This was the effect of reduced association between anxiety and heart-IR in these clusters on citalopram. A visual appraisal of the data indicated that the heart-IR within these regions increased with state anxiety when participants were on a placebo, but this relationship was reversed on citalopram (Fig. 4B). This was confirmed in a follow-up independent whole brain cluster-corrected analysis of the placebo condition. State anxiety increased with heart-IR in the right anterior insular/orbitofrontal cortex (316 voxels, Peak Z = 5.13, MNI coord: 44, 30, −12). On the left, this effect was also present but did not survive cluster correction (58 voxels, *p* < 0.001 uncorrected, peak MNI coord −44, 20, 8). No such effect was present on citalopram (Fig. 4A, Table S2). Further associations between state anxiety and heart-IR were observed on a placebo, but only the anterior insular/orbitofrontal clusters demonstrated a reduced association on citalopram. A similar anxiety-dependent effect was not observed for stomach-IR.

The state anxiety measure captured anxiety during the scan, which medication side effects could influence. The state anxiety-by-treatment interaction was robust to covariates of nausea, dizziness, headache, guess of drug condition, and the interactions of these measures with the treatment condition (see Supplemental Material). However, to further rule out the influence of side effects, we tested and found that trait anxiety (STAI-T), measured before any treatment and therefore unrelated to side effects, also predicted the effect of citalopram on heart-IR within the same bilateral orbitofrontal/insular cortex clusters (Table S3, Figure S2). This supported the conclusion that this treatment effect on heart-IR relates to levels of anxiety rather than drug-induced correlates.

**Fig. 5:**
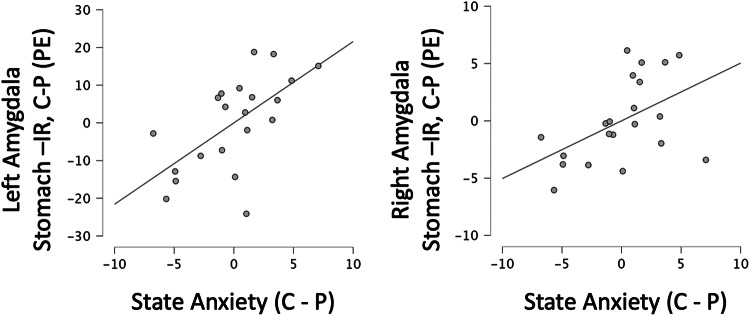
Association between Citalopram Effects on State Anxiety and Stomach-IR. Change in state anxiety corelated with change of stomach-IR (extracted average contrast parameter estimates in stomach-IR amygdalar clusters (Table 1, Fig. 2). C-P is citalopram – placebo. Right Amy = amygdalar cluster.

Changes in state anxiety were associated with changes in nausea (*r* = 0.68, *p* < 0.001), headache (*r* = 0.54, *p* = 0.014), and dizziness (*r* = 0.45, *p* = 0.047) after controlling for treatment order. In the post-hoc examination of the main effects of heart-IR and stomach-IR clusters, the variance in state anxiety change after controlling for these associations was associated with treatment differences within the average extracted parameter estimates of the left amygdala cluster (b = 2.2, t(15) = 3.1, *p* = 0.008, r = 0.62) and right amygdala cluster (b = 0.5, t(15) = 2.2, *p* = 0.044, *r* = 0.49) of the stomach-IR (Fig. 5). A Bonferroni corrected alpha level for this post hoc analysis (6 tests) is *p* = 0.0083. The right amygdala effect is reported here due to the same effect on the left.

### Metacognitive Interoceptive Insight

In a previous study involving contributions from the same participants, a single citalopram dose increased conscious awareness of the reliability of inferences made from heartbeat interoception [16]. We explored whether this effect correlated with the SSRI-induced reduction of heart-IR, which it did within the left (but not right) amygdala (see Supplemental Material, Figure S1).

### Robustness tests

For a thorough examination of the robustness of effects to treatment side effects, treatment order, and guess of treatment condition, see Supplemental Material.

## Discussion

A single dose of the SSRI citalopram reduced the relative neural response to attended normal internal sensation in viscerosensory (e.g. posterior insular cortex) and integrative/emotion-processing regions (e.g. amygdala) of interoceptive processing pathways [3, 78]. The posterior insular cortex, in which citalopram reduced stomach-IRs, is distinct from mid or anterior insular regions in its connectivity and cytoarchitecture [3, 79]. It is the predominant destination for topographically organised viscerosensory information from the thalamus, originating from ascending sympathetic and parasympathetic pathways from body tissues [1, 49, 79]. Neuroimaging studies demonstrate reliable activation of this insular region by gastric sensation [31, 80], suggesting that increased extracellular serotonin reduced primary sensory processing of the upper gastrointestinal tract. Theoretically, this basic sensory information then travels through hierarchical networks to the mid and anterior regions of the insular cortex, where it is integrated into conceptual interoceptive representations through interaction with the prefrontal cortex, amygdala and striatum to influence allostasis, motivation, emotion, and related cognitive processes [3, 49, 78, 81].

In the amygdalae, both stomach-IRs and heart-IRs were reduced by citalopram. The amygdalae are innervated by serotonin projections from the raphe nuclei and receive interoceptive information via ascending viscerosensory pathways of the brainstem, the thalamus, and interconnections with the cortex (strongly connecting with anterior insula cortex) [79, 80, 82, 83]. Here, the interoceptive information is proposed to steer allostasis by influencing arousal and salience attribution [34, 78, 81, 84]. In the present study, the reduction of IRs could reflect reduced interoceptive input to the amygdala or a change in how the amygdala uses interoceptive sensations.

Some effects of citalopram were state-dependent. Prior research demonstrated that individuals with greater anxiety exhibit a greater right [44] and left [45] anterior insula cortex response to attended heart sensation. In the present study, we confirmed this state-dependent relationship in the placebo condition in the anterior insular cortex (overlapping on the left but ventral to previous findings on the right). Heart-IR in the anterior insular and adjoining orbitofrontal cortex was reduced by citalopram in proportion to anxiety levels, with a significant flattening of the anxiety-interoception relationship observed on placebo. Critically, this effect represents a reduction in the link between anxiety and interoception, not an anxiety reduction. Reduced links between anxiety and behavioural measures of interoception have been previously associated with increased anhedonia [42]. One might, therefore, wish to explore whether SSRI-induced changes in the neural anxiety-interoception relationship in the anterior insula relate to emotional blunting associated with SSRI treatment in some individuals [40]. Moreover, anxious states have been proposed to relate to accumulating, reciprocal relationships between levels of anxiety, heightened interoceptive responses, and further anxiety about those heightened interoceptive responses [27, 39]. So, our finding also sets the stage for investigations of whether serotonergic perturbation of the anxiety-interoception relationship could have a role in long-term SSRI treatment outcomes. More generally, however, this result confirms that some modulatory effects of serotonin on ordinary interoceptive processing are state-dependent, as previously suggested for serotonin influence on exteroceptive processes [9, 56–58].

As expected from prior research, anxiety responses increased for some participants and decreased for others, resulting in no net change [25, 39, 50]. We have provided preliminary insight into the individual differences in anxiety response, suggesting that the amygdala’s response to stomach sensation relates to increases and decreases in anxiety following an SSRI dose. The bilaterality of the effect provides confidence in this result. If replicated in a larger sample, this effect would underscore the importance of considering links between anxiety and gastrointestinal sensation in psychiatric and internal medicine research [29, 85, 86].

Like similar experimental medicine studies, we employed a single-dose SSRI protocol to understand the cognitive impact of acutely increased endogenous serotonin [39, 50]. Acute effects of SSRIs can differ from effects after seven or more days due to desensitisation of autoreceptors and other adaptive effects [67, 87]. Additional research would therefore be needed to extrapolate to the longer-term effects of SSRI treatment. Secondly, this was a study of young, healthy volunteers. This provides controlled inferences about normal function unencumbered by interactions with symptoms or other medications and avoids ethical challenges of within-subject pharmacological study in a patient group. However, only with further research should one extrapolate these findings to clinical contexts and experiences across the lifespan. There is also much more to learn about the precise mechanisms of these effects. Citalopram has exceptional selectivity for the serotonin transporter. However, the role of knock-on effects on other neurotransmitter systems and the roles of specific serotonin receptors remains unknown.

Overall, we found that an acute increase in extracellular serotonin reduces central neural responses to interoceptive information in healthy individuals in both general and anxiety-dependent fashion. This opens new avenues of research in other populations and contexts for a better understanding of interoception, its regulation, and its relationship to affective states.

### Supplementary information

Supplemental Material
