# Supplementary material for: General and anxiety-linked influences of acute serotonin reuptake inhibition on neural responses associated with attended visceral sensation
Source: Transl Psychiatry. 2024 Jun 6;14:241. doi: 10.1038/s41398-024-02971-3 (PMC11156930; doi:10.1038/s41398-024-02971-3)
Supplement: Supplementary file 1 — Supplemental Material [file 41398_2024_2971_MOESM1_ESM.pdf]

Supplemental Material

Title: General and Anxiety-Linked Influences of Acute Serotonin Reuptake Inhibition on Neural Responses Associated with Attended Visceral Sensation

Authors: James J A Livermore PhD[1], Lina I Skora PhD [1,2,3], Kristian Adamatzky PhD[1], Sarah N Garfinkel PhD [5], Hugo D Critchley FRCPsych, DPhil [3,4,6], and Daniel Campbell-Meiklejohn DPhil[1]

Affiliations:

1 School of Psychology, University of Sussex, Brighton, UK
2 Heinrich Heine Universität, Düsseldorf, Germany
3 Sussex Centre for Consciousness Science, University of Sussex, Brighton, UK
4 Brighton and Sussex Medical School, Brighton, UK
5 Institute of Cognitive Neuroscience, University College London
6 Sussex Partnership NHS Foundation Trust, UK

All work was completed at the University of Sussex.

Corresponding Author

Dr Daniel Campbell-Meiklejohn
School of Psychology
Pevensey Building
University of Sussex
Falmer, East Sussex
BN1 9QH
dc307@sussex.ac.uk

Keywords

Interoception, Serotonin, Anxiety

Table of Contents

SUPPLEMENTAL RESULTS.......... 3

CEREBRAL BLOOD FLOW.......... 3

TABLE S1: HEART RATE AND SUBJECTIVE EXPERIENCE.......... 4

TABLE S2: FMRI EFFECT CLUSTERS OF STAI-S ASSOCIATION WITH HEART-IR IN PLACEBO CONDITION.......... 4

RELATIONSHIPS BETWEEN CLUSTERS.......... 5

CARDIAC INTEROCEPTIVE INSIGHT.......... 5

Figure S1. Change in Metacognitive Cardiac Interoceptive Insight and Neural Response to Heart Focus in the Left Amygdala.......... 6

ROBUSTNESS TESTS.......... 7

CONSIDERING CITALOPRAM'S INTERACTION WITH STATE AND TRAIT ANXIETY.......... 7

Figure S2. Effect of Citalopram on the 'Trait' Anxiety-Interoception Relationship.......... 9

Table S3. Trait Anxiety (STAI-T) x Citalopram Effect on Heart-IR.......... 10

CONSIDERING HEARTRATE.......... 10

CONSIDERING SESSION ORDER.......... 10

CONSIDERING GUESS OF THE TREATMENT CONDITION.......... 11

ROBUST DATASET RESULTS.......... 11

Figure S3. Self-Stated Probability of Drug Condition, Robust Dataset (N=16).......... 12

Figure S4. Citalopram's Effect on Stomach Focus, Robust Dataset (N=16).......... 13

Table S4. Citalopram's Effect on Stomach Focus, Robust Dataset (N=16).......... 13

Figure S5. Citalopram's Effect on Heart Focus, Robust Dataset (N=16).......... 14

Table S5. Citalopram's Effect on Heart Focus, Robust Dataset (N=16).......... 14

Figure S6. Citalopram's Effect on Heart Focus x State Anxiety, Robust Dataset (N=16).......... 15

Table S6. Citalopram's Effect on Heart Focus x State Anxiety, Robust Dataset (N=16).......... 15

Figure S7. Association Between Citalopram's Effect on State Anxiety and Neural Response to Stomach Focus (stomach-IR), Robust Dataset (N=16).......... 16

Figure S8. Association Between Citalopram's Effect on Metacognitive Cardiac Interoceptive Insight and Neural Response to Heart Focus (heart-IR), Robust Dataset (N=16).......... 16

Supplemental Results

Cerebral Blood Flow

Changes in resting perfusion that may accompany the pharmacological treatment and confound task-related changes in BOLD signal were measured with pulsed arterial spin labelling (ASL) images acquired using a FAIR-QII sequence (4 label-control image pairs, echo time 16ms, repetition time 4600ms, voxel size 1.5 × 1.5 × 3mm, 126 × 128 voxels per slice, 40 slices, interleaved slices, field of view 192 × 192mm^2, flip angle 180°, inversion time 1990ms, bolus duration 700ms).

ASL images were processed using Bayesian Inference for Arterial Spin Labelling to produce perfusion-weighted images. ASL images were analysed at the group level in a paired t-test of CITALOPRAM and PLACEBO sessions using Statistical Parametric Mapping (SPM12), with a cluster-wise family-wise error rate of $p < .05$, and with an additional exploratory threshold of $p < .001$ with extent > 50 voxels per cluster.

Analysis of ASL image pairs showed no significant clusters at the familywise error rate, suggesting that CITALOPRAM did not affect cerebral blood flow. Therefore, any effects on BOLD responses were unlikely to be mediated by general effects on blood flow in the brain. With an uncorrected threshold, a single significant cluster was shown in the occipital cortex (peak voxel MNI coordinate: (18, -96, 14), $p(uncorrected) = .001$, $pFWE = .10$).

Table S1: Heart Rate and Subjective Experience

	CITALOPRAM		PLACEBO		*Difference*	
	Mean	*SD*	*Mean*	*SD*	*t(20)*	*p*
Task Intensity Rating						
Heart	2.29	0.51	2.16	0.53	1.10	0.29
Stomach	1.85	0.51	1.75	0.64	0.96	0.35
Visual Target	2.44	0.45	2.31	0.33	1.20	0.25
Heart Rate						
Heart Rate (bpm)	63.10	8.44	66.53	10.62	-2.45	0.02
Heart Rate Variability (bpm SD)	2.27	1.31	2.73	1.25	-1.02	0.32
VAS Scales (1-100)						
Nausea	8.98	11.08	4.43	6.40	1.56	0.14
Dizziness	15.33	15.23	11.88	12.00	1.01	0.32
Headache	11.14	15.98	13.12	18.22	-0.58	0.57
Alert−Drowsy	56.88	19.88	52.24	17.09	0.77	0.45
Stimulated−Sedated	52.95	18.08	51.38	17.56	0.28	0.79
Restless−Peaceful	60.00	20.88	63.24	23.73	-0.62	0.54
Irritable−Good humoured	64.05	16.84	64.26	19.43	-0.07	0.94
Anxious−Calm	66.19	19.54	71.57	22.70	-1.30	0.21
State Anxiety (STAI-S)	38.52	10.27	38.12	11.63	0.34	0.73
PANAS-Negative	13.50	3.62	13.14	4.38	0.45	0.66
PANAS-Positive	20.74	5.78	20.02	5.70	0.56	0.58

Table S2: fMRI Effect Clusters of STAI-S Association with Heart-IR in Placebo Condition. Contrast, region (Oxford Harvard Cortical and Subcortical Atlas), number of voxels in cluster, peak voxel z score and peak coordinates (x,y,z) in MNI space. Cluster-corrected analysis (Z >3.1, p < 0.05).

Anatomy	***Voxels***	***Z MAX***	***X***	***Y***	***Z***
Superior Frontal Gyrus	721	5.97	10	36	54
Frontal Pole	635	5.38	42	44	-4
Cerebellum	338	5.03	0	-76	-24
Frontal Orbital Cortex / Anterior Insular Cortex	316	5.13	44	30	-12
Supramarginal Gyrus	306	5.61	50	-42	56
Middle Temporal Gyrus	270	4.74	66	-30	-4
Cerebellum	266	5	-44	-64	-48
Cerebellum	114	4.48	-4	-42	-18
Cerebellum	101	4.58	24	-34	-26

Relationships Between Clusters

Across participants, average posterior insula cluster responses to CITALOPRAM while focusing on the stomach, on the left and right, were highly correlated, sharing 84% of their variance (r = .92, p < 0.001). Amygdala clusters during stomach focus were less correlated with each other (r = 0.57, *p* = 0.007) and with insula responses (left amygdala cluster / left posterior insula cluster: r = .56, r = .47, *p* = 0.03; right amygdala cluster / right post insula cluster: r = .58, *p* = 0.006), each sharing around 32% of variance. Similarly, during heart focus, amygdala cluster changes shared around 28% of the variance (r = .53, *p* = .014).

Cardiac Interoceptive Insight

Participants also performed an independent heartbeat discrimination task outside the scanner (for a full description of this task and protocol see [1]). During each of 20 trials (performance on 20 trials correlate at r = .7 with performance on 100 trials [2]), their heartbeat was measured in real-time, while a computer played a set of ten tones at either 250 ms or 550 ms after the R-wave corresponding to judgements of maximum and minimum simultaneity. The participant was directed to respond to whether the tones were in or out of time with their heartbeats and how confident they were in that answer using a VAS scale ranging from 'total guess' to 'complete confidence' on a scale of 1 to 10. Metacognitive cardiac interoceptive insight is measured as the ability of confidence to classify correct and incorrect responses. In a large sample to which this project contributed, CITALOPRAM increased this ability [1]. CITALOPRAM's effect on cardiac interoceptive insight was tested for association with neural effects during heart focus of the VIA task (Figure 3) for participants who performed both tasks. One participant of the present dataset was removed due to feeling their pulse in the finger. Change of activity within the left, but not right, amygdalar cluster on CITALOPRAM was associated with increased interoceptive insight (r = -.48, *p* = 0.033). This effect remains after controlling for change of heart rate (r = -.55, *p* = .016) or treatment order (r = -.47, *p* = 0.039), *p* = .021) (Figure S1).

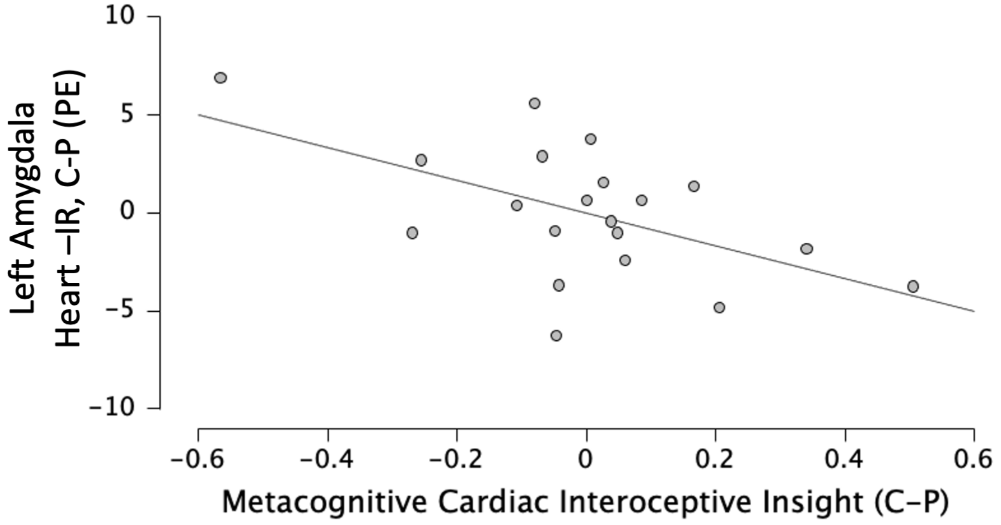

Figure S1. Change in Metacognitive Cardiac Interoceptive Insight and Neural Response to Heart Focus in the Left Amygdala. C = CITALOPRAM, *P* = PLACEBO. This partial residual plot indicates the relationship between CITALOPRAM's effect on metacognitive interoceptive insight and the left amygdala heart-IR (VIA task, Figure 3) after controlling for treatment order and changes in heart rate.

Robustness Tests

CONSIDERING CITALOPRAM's Interaction with State and Trait Anxiety

On one hand, the interaction effect of CITALOPRAM generating a greater *reduction* of heart-IR in the anterior insular/orbitofrontal cortex with greater anxiety would be unlikely to be caused by anxiety-generating side effects because increased visceral sensation from side effects such as nausea would be expected to increase the neural response in these regions. Only an association between state anxiety and dizziness was found (dizziness r = .38, *p* = .015; nausea r = .251, *p* = .118; headache r = .226, *p* = .160) and only if uncorrected for multiple comparisons. On the other hand, one may also avoid attending visceral sensations after initially experiencing side effects. This could theoretically result in a reduction of heart-IR. So, to test for complete independence of these side effects, we constructed linear mixed models of heart-IR using extracted parameter estimates from significant orbitofrontal/anterior insular clusters (Figure 3). These models included random intercepts and fixed effects of treatment (PLACEBO/CITALOPRAM), session, STAI-S scores, treatment x STAI-S interaction, and main effects of nausea, headache, dizziness and each of their interactions with treatment. For both clusters, the STAI-S x treatment interaction remained strongly significant (*ps* < .001), despite covariates.

While STAI-S best captures anxiety levels at that moment, the trait anxiety measure, STAI-T, captures how one *generally* feels. STAI-T scores were measured before any treatment was received and, therefore, unaffected by the side effects of the drug. STAI-T scores were not related to headache, nausea, or dizziness (*p*s > .3). If the interaction effect of STAI-T x treatment overlaps with the STAI-S x treatment interaction effect on heart-IR, this would be further assurance that the latter interaction is driven by anxiety and not side effects of the SSRI. A linear mixed model (random intercept, fixed effects of treatment, STAI-T scores, their interaction, and treatment order) demonstrated that trait anxiety scores moderated the effect of CITALOPRAM in the right (*b* = -3.39, t(21) = -4.4, p < .001) and left (*b* = -1.7, t(21) = -3.1, *p = .*003) frontal orbital cortex / insular cortex clusters of Figure 3, similar to STAI-S scores. These

effects were unaffected by the addition of nausea, headache, dizziness and/or their interaction with CITALOPRAM as additional fixed effects, and these latter effects were not significant.

For even further assurance, the interaction of trait anxiety (STAI-T) and CITALOPRAM was investigated as a parametric predictor of CITALOPRAM effects, with a three-level analysis in FSL. Lower-level analyses were contrasted within each subject at a second level to generate contrast images for each participant that represented differences in activity between treatment conditions. These contrast images were then elevated to a third-level analysis, which allowed for the test of association of differences with variation in STAI-T scores, entered as a mean-centred covariate, together with a variable of treatment order. In a whole-brain search, STAI-T scores predicted drug effects in the same right anterior insular/orbitofrontal cortex cluster on the right as STAI-S. If statistical cluster correction is limited to regions of CITALOPRAM effects associated with state anxiety (STAI-S), the association with trait anxiety (STAI-T) is also present on the left. See Figure S2.

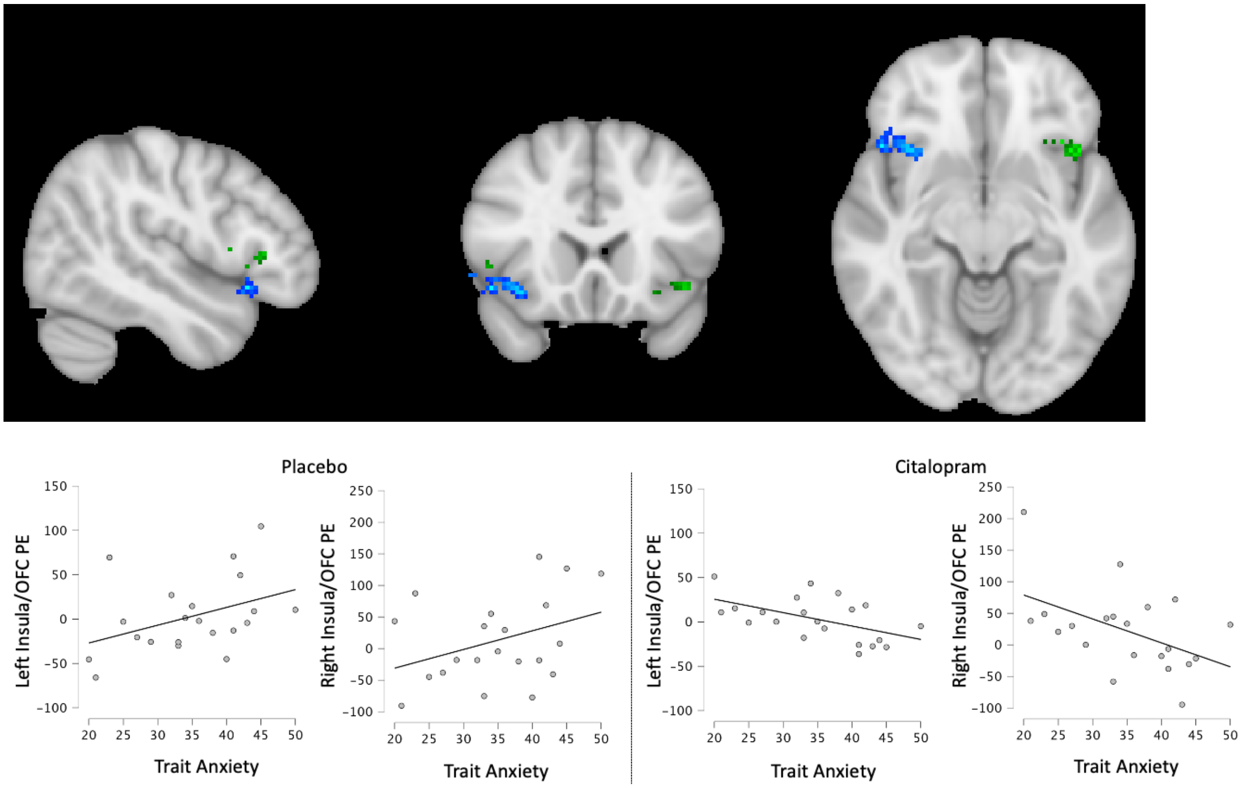

Figure S2. Effect of Citalopram on the 'Trait' Anxiety-Interoception Relationship: Top: Reduction of relative neural interoceptive response to heart sensation (heart-IR) following 20mg of CITALOPRAM (< PLACEBO) in proportion to trait anxiety (STAIT-T), measured before any treatment. Blue maps are whole brain cluster corrected. The green map resulted from small volume correction within clusters of equivalent contrast using state anxiety (STAI-S) (Figure 4, Table 1). Maps are developed by a voxel threshold of $Z > 3.1$ and a cluster significance threshold of $p < 0.05$. $N = 21$. **Bottom:** Scatter plots of STAI-T scores to heart-IR in CITALOPRAM and PLACEBO conditions within frontal orbital cortex / insular clusters generated by the STAI-S x treatment interaction (Figure 4, Table 1). Illustration only. Use Top and Table S3 for statistical inference.

Table S3. Trait Anxiety (STAI-T) x Citalopram Effect on Heart-IR. *Harvard-Oxford Atlas label, cluster size, Z score of peak voxels, and MNI coordinates.*

	Voxels	Z MAX	X	Y	Z
Whole brain search:					
Frontal Orbital Cortex / Insular Cortex	186	4.29	48	20	-10
Frontal Pole	157	4.29	20	52	22
Small volume correction, within STAI-S x CITALOPRAM effect clusters:					
Frontal Orbital Cortex / Insular Cortex	119	4.29	48	20	-10
Frontal Orbital Cortex / Insular Cortex	59	4.27	-40	18	-10
Inferior Frontal Gyrus	22	4.05	52	14	8
Inferior Frontal Gyrus	18	3.81	48	28	6
Precuneus	7	3.92	6	-82	50
Lateral Occipital Cortex	5	3.55	-26	-82	-2

Considering Heartrate

Given that CITALOPRAM reduced heart rate, it was important to understand whether this influenced our results. We ran a *post hoc* repeated measures mediation analysis to test whether the effect of CITALOPRAM on each of the reported responses (extracted average contrast parameter estimates from Table 1) is independent of heart rate changes. This includes a test of the drug effect in the presence of a covariate of 'change in heart rate'. The direct effect of CITALOPRAM on neural responses was preserved in all cases ($p < 0.001$); there was no interaction of drug effects with the heart rate change across participants and no mediation by the heart rate change.

Considering Session Order

All mixed-effect model imaging results include a covariate for the effect of whether it was the participant's first or second time performing the interoception task in the scanner. In addition, a separate analysis of order effects did not demonstrate any significant effect overlapping with the effects of CITALOPRAM.

However, after participant withdrawals, the sample was not balanced between randomised treatment order conditions: thirteen received CITALOPRAM in the first session, and eight participants received a PLACEBO in the first session. We sought certainty that this did not influence inferences made from our results. We, therefore, addressed this with a robust dataset with a perfect balance of treatment order, in which all results were preserved (see below).

Considering Guess of the Treatment Condition

To indirectly capture the somatic effects of CITALOPRAM and test the preservation of the blind, we asked participants to rate their belief that they had received CITALOPRAM in each session at the time of testing. No participant correctly responded with a certainty of being on CITALOPRAM or PLACEBO. However, a paired t-test demonstrated that the rated probability of being on CITALOPRAM was higher when participants had received CITALOPRAM ($t(20) = 2.42$, $p = 0.03$). This is an imperfect measure because the second session ratings result from different information than the first. The difference between CITALOPRAM and PLACEBO condition self-rated probabilities of being on the drug were higher if CITALOPRAM was received first ($t(19) = 2.3$, $p = .035$). Since the experimenter was blind to the conditions, the increased rated probability of being on CITALOPRAM after receiving it was unlikely due to implicit cues from the experimenter. Regardless, this effect merited further investigation in the robust dataset below.

Robust Dataset Results

To address the potential effects of treatment order and insight into drug conditions, we developed a supplementary robust dataset. This group was created by removing participants in the larger treatment order group with the most significant difference of belief of having received CITALOPRAM between drug conditions until the groups were balanced for order (8 participants in each order) and guess of treatment ($t(15) = 0.86$, $p = .40$), with a total of 32 test sessions (Figure S3).

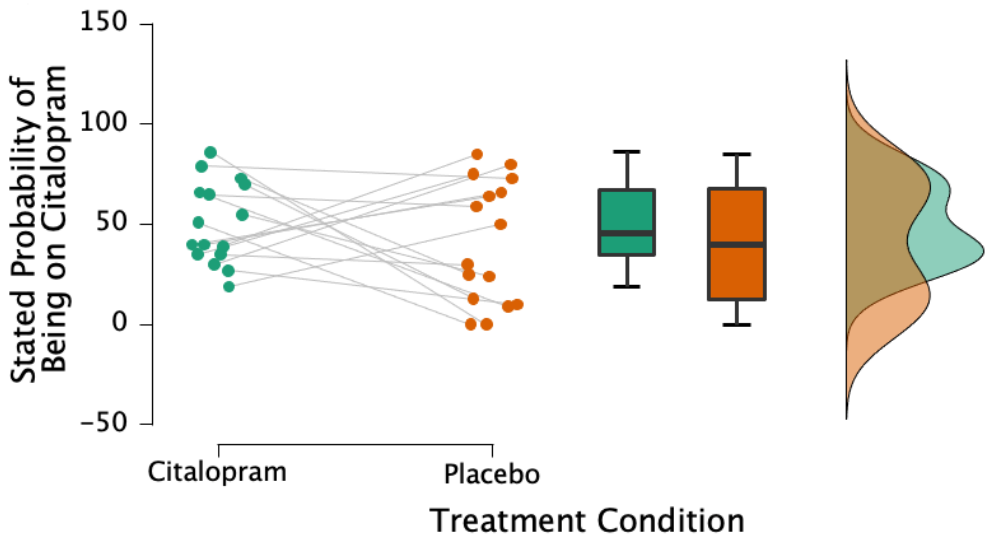

Figure S3. Self-Stated Probability of Drug Condition, Robust Dataset (N=16).

Using this robust dataset, there was no difference in any subjective measure between treatment conditions (*p*s > .25). Near identical results were found using this robust dataset as found for the full dataset (Figures S4 to S8, Tables S4 to S6). The only effect losing significance (but remaining on trend) was the association of anxiety change to the right amygdala cluster response to CITALOPRAM (left amygdala cluster effect remains). Therefore, in general, fMRI effects could not be attributed to treatment order or belief in drug conditions. See below for Tables and Figures relating to the replication of contrasts using the robust dataset.

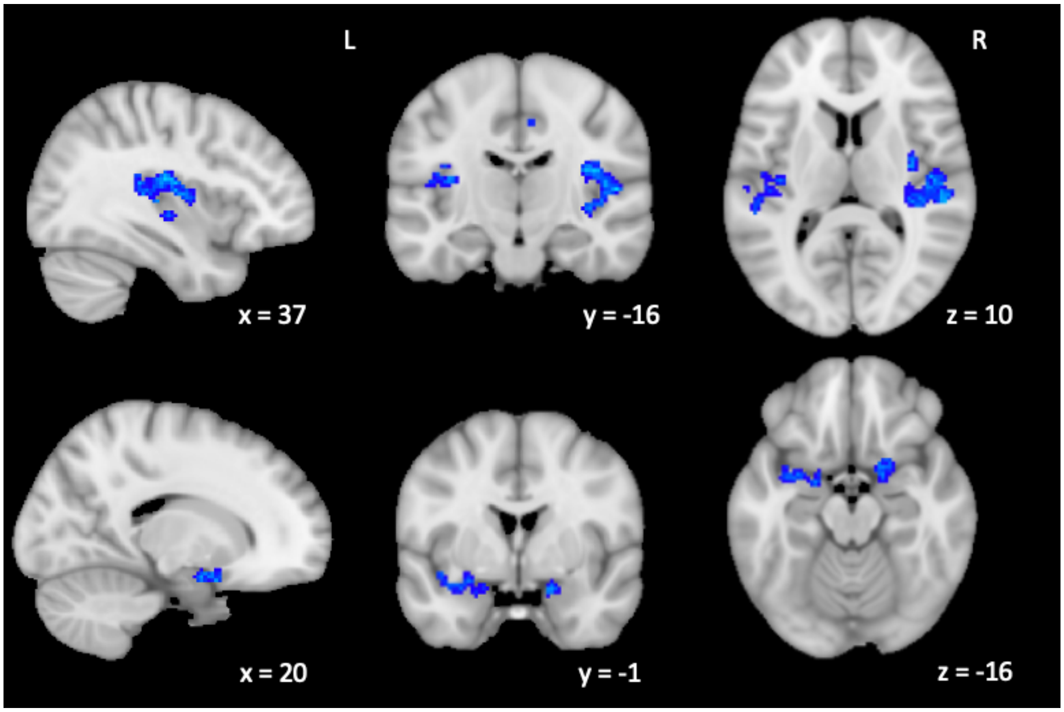

Figure S4. Citalopram's Effect on Stomach Focus, Robust Dataset (N=16). Reduced relative activation following 20mg of CITALOPRAM (compared to PLACEBO) while attending to the stomach (stomach-IR). Deactivation maps overlayed onto the standard MNI 152 brain (coordinates indicated) and developed with a voxel threshold of Z > 3.1, and a cluster significance threshold of $p < 0.05$. Maps generated after controlling for treatment order. R = Right, L = Left. Compare to Figure 2.

Table S4. Citalopram's Effect on Stomach Focus, Robust Dataset (N=16). Harvard-Oxford Atlas label, cluster size, Z score of peak voxels, and MNI coordinates.

REGION	VOXELS	Z MAX	X	Y	Z
Right posterior insula cluster: right planum temporale peak, extending to Heschl's gyrus, posterior insular cortex, central opercular cortex	*742*	*5.13*	*40*	*-34*	*14*
Left posterior insula cluster: left posterior insular cortex peak extending to Heschl's gyrus, insular cortex, central opercular cortex and planum temporale	*385*	*4.71*	*-36*	*-18*	*14*
precentral gyrus	*303*	*4.62*	*10*	*-26*	*46*
Left amygdala cluster: frontal orbital cortex peak extending to left amygdala and anterior insular cortex	*184*	*4.75*	*-24*	*8*	*-22*
cerebellum	*153*	*4.87*	*-8*	*-76*	*-40*
cerebellum	*123*	*4.34*	*-10*	*-50*	*-54*
Right amygdala cluster: right frontal orbital cortex extending to right amygdala	*105*	*4.43*	*20*	*6*	*-20*

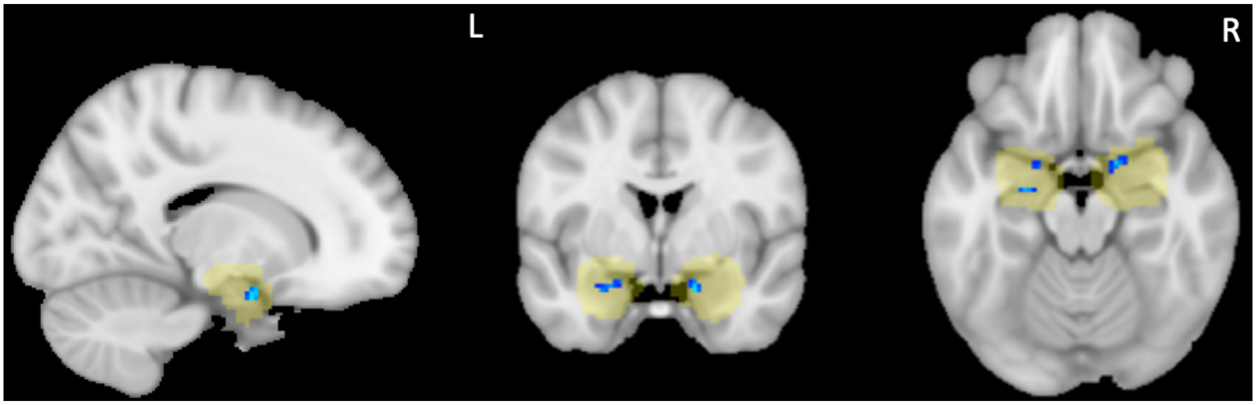

Figure S5. Citalopram's Effect on Heart Focus, Robust Dataset (N=16). A. Reduced relative activation in the amygdala following 20mg of CITALOPRAM (vs. PLACEBO) while attending to the heart (heart-IR). Deactivation maps (blue) were overlayed onto the MNI 152 brain (x = 16, y = 0, z = -18). Deactivation maps overlayed onto the standard MNI 152 brain, developed by a voxel threshold of Z > 3.1, a cluster significance threshold of $p < 0.05$, and small volume correction within an anatomical mask covering any probability of amygdalae using the Harvard-Oxford Subcortical Structural Atlas (yellow). R-L indicate right and left. Compare to Figure 3.

Table S5. Citalopram's Effect on Heart Focus, Robust Dataset (N=16). Harvard-Oxford Atlas label, cluster size, Z score of peak voxels, and MNI coordinates.

**Region**	**Voxels**	**Z MAX**	**X**	**Y**	**Z**
right amygdala	38	4.02	16	0	-22
	30	4.15	24	-10	-22
left amygdala	27	3.77	-24	-10	-18
	19	3.75	-24	2	-22

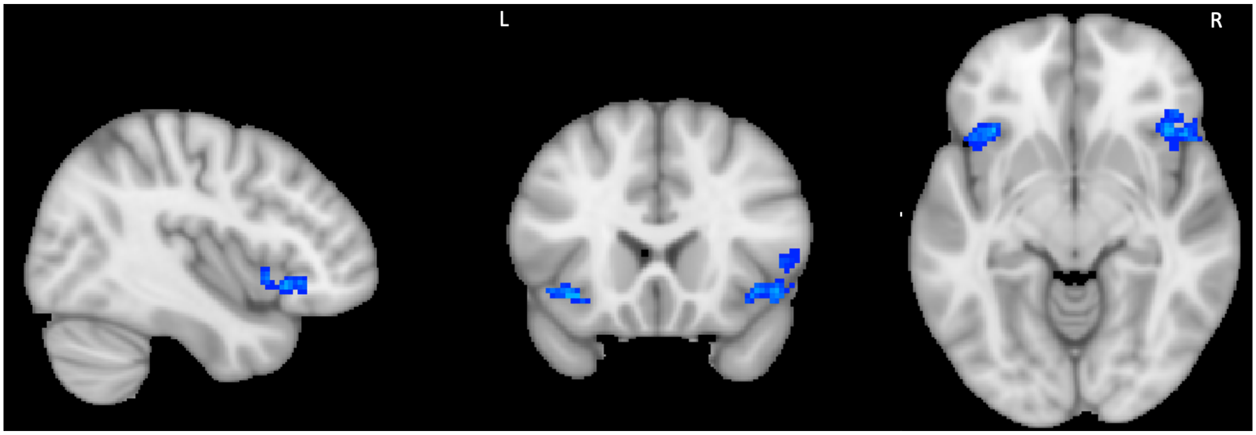

Figure S6. Citalopram's Effect on Heart Focus x State Anxiety, Robust Dataset (N=16). Reduced relative activation following 20mg of CITALOPRAM (compared to PLACEBO), while attending to the heart (relative to visual stimuli) (heart-IR) in proportion to state anxiety. Deactivation maps overlayed onto the MNI 152 brain (x = 41, y = 20, z = -8) and developed by a voxel threshold of Z > 3.1, and a cluster significance threshold of $p < 0.05$. Compare to Figure 4A.

Table S6. Citalopram's Effect on Heart Focus x State Anxiety, Robust Dataset (N=16). Harvard-Oxford Atlas label, cluster size, Z score of peak voxels, and MNI coordinates.

**Region**	**Voxels**	**Z MAX**	**X**	**Y**	**Z**
frontal pole, right	296	4.97	28	58	24
frontal orbitofrontal cortex / insular cortex, right	261	4.42	40	22	-8
lateral occipital cortex, superior division, right	143	4.61	50	-70	32
frontal pole, left	121	4.61	-20	52	36
frontal orbitofrontal cortex / insular cortex, left	99	4.5	-34	22	-8
superior frontal gyrus, right	88	4.01	4	28	58

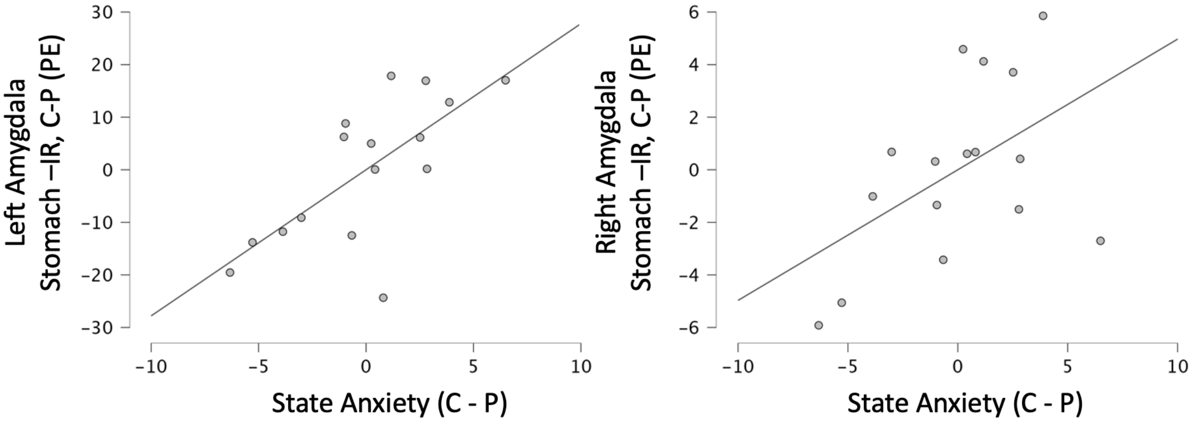

Figure S7. Association Between Citalopram's Effect on State Anxiety and Neural Response to Stomach Focus (stomach-IR), Robust Dataset (N=16). Partial residual regression plots, after controlling for treatment order, change of nausea, change of headache and change of dizziness. C-P is CITALOPRAM – PLACEBO. Left amygdala cluster ($b = 2.7$, $t(10) = 3.09$, $p = .011$), right amygdala cluster ($b = .50$, $t(10) = 1.8$, $p = .096$). Compare to Figure 5.

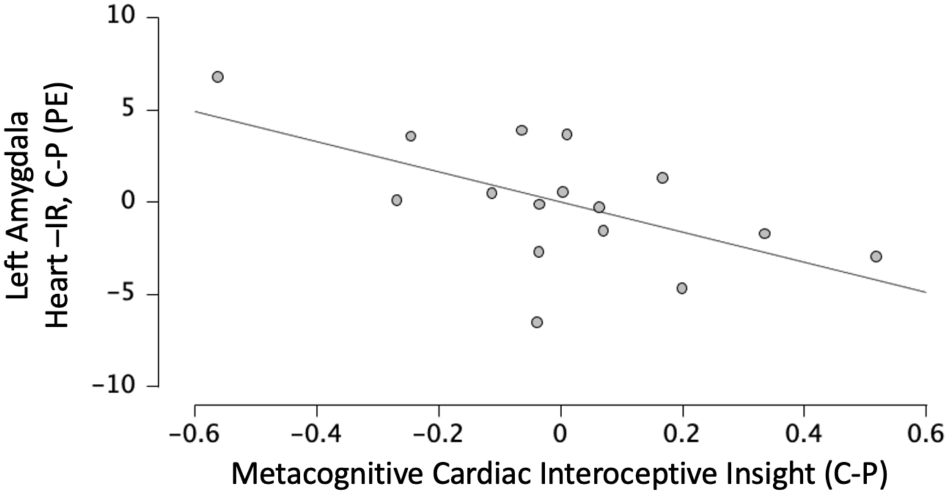

Figure S8. Association Between Citalopram's Effect on Metacognitive Cardiac Interoceptive Insight and Neural Response to Heart Focus (heart-IR), Robust Dataset (N=16). Partial residual regression plot after controlling for treatment order and change of heart rate. C - P is CITALOPRAM – PLACEBO. Left amygdala cluster: ($b = -8.15$, $t(12) = -2.5$, $r = -.59$, $p = .025$). Compare to Figure S1.
